# Recent Advances and Impact of Chemotherapeutic and Antiangiogenic Nanoformulations for Combination Cancer Therapy

**DOI:** 10.3390/pharmaceutics12060592

**Published:** 2020-06-25

**Authors:** Amit Kumar Rajora, Divyashree Ravishankar, Hongbo Zhang, Jessica M. Rosenholm

**Affiliations:** 1 Pharmaceutical Sciences Laboratory, Faculty of Science and Engineering, Åbo Akademi University, 20520 Turku, Finland; hongbo.zhang@abo.fi; 2 Bioscience Department, Sygnature Discovery, Bio City, Pennyfoot St, Nottingham NG1 1GR, UK; d.ravishankar@sygnaturediscovery.com; 3 Turku Bioscience Center, University of Turku and Åbo Akademi University, 20520 Turku, Finland

**Keywords:** anticancer, antiangiogenic agents, nanoformulations, combination cancer therapy

## Abstract

Traditional chemotherapy, along with antiangiogenesis drugs (combination cancer therapy), has shown reduced tumor recurrence and improved antitumor effects, as tumor growth and metastasis are often dependent on tumor vascularization. However, the effect of combination chemotherapy, including synergism and additive and even antagonism effects, depends on drug combinations in an optimized ratio. Hence, nanoformulations are ideal, demonstrating a great potential for the combination therapy of chemo-antiangiogenesis for cancer. The rationale for designing various nanocarriers for combination therapy is derived from organic (polymer, lipid), inorganic, or hybrid materials. In particular, hybrid nanocarriers that consist of more than one material construct provide flexibility for different modes of entrapment within the same carrier—e.g., physical adsorption, encapsulation, and chemical conjugation strategies. These multifunctional nanocarriers can thus be used to co-deliver chemo- and antiangiogenesis drugs with tunable drug release at target sites. Hence, this review attempts to survey the most recent advances in nanoformulations and their impact on cancer treatment in a combined regimen—i.e., conventional cytotoxic and antiangiogenesis agents. The mechanisms and site-specific co-delivery strategies are also discussed herein, along with future prospects.

## 1. Introduction

Cancer is one of the leading causes of death, and its treatment remains one of the most severe challenges worldwide. Globally, about 1 in 6 deaths is due to cancer. The World Health Organization (WHO) projected that cancer was responsible for an estimated 9.6 million deaths in the year 2018. The number of global cancer deaths is currently projected to increase by 45% between 2008 and 2030 [1]. Carcinogenesis is a complex and dynamic process, comprised of cancer-associated fibroblasts and myofibroblasts, neuroendocrine cells, adipose cells, immune and inflammatory cells, blood and lymphatic vascular networks, and an extracellular matrix (ECM). These processes altogether establish a complex cross-talk within the tumor microenvironment. Hence, cancer is described as a group of diseases characterized by uncontrolled growth/proliferation and the spread of abnormal cells [2,3,4,5,6]. Conventional treatment approaches for cancer include surgery; radiotherapy; and systemic treatments such as chemotherapy, endocrine therapy, and antiangiogenic therapy [7,8,9,10]. This paper will present the recent nanoformulation developments of anticancer (chemotherapy) and antiangiogenesis agents.

Chemotherapy for cancer treatment involves the use of various anticancer drugs with different mechanisms of action, viz, preventing cell division, triggering apoptosis, and targeting the cancer cells [11,12,13,14]. Though chemotherapy has gained widespread momentum in cancer treatment, the adverse drug reactions outweigh the therapeutic efficacy. On the other hand, antiangiogenic agents act on tumor blood vessels and block tumor growth and malignancy [10,15]. Angiogenesis is a physiological process through which new blood vessels form from pre-existing vessels [16,17,18,19,20]. Tumor growth requires a sufficient supply of oxygen and nutrients to support their further growth and metastasis and a variety of growth factors secreted by tumors, such as vascular endothelial growth factor (VEGF), matrix metalloproteinase (MMP), epidermal growth factor (EGF), and platelet-derived growth factor (PDGF) [17]. Among the abovementioned growth factors, VEGF is the most important proangiogenic cytokine that regulates angiogenesis. VEGF binds to its receptor (VEGFR) and activates the receptor that triggers downstream signals, which subsequently promote the proliferation, migration, and tube formation of endothelial cells, finally promoting angiogenesis [17,21,22,23,24]. Hence, the inhibition of tumor angiogenesis by interfering with the VEGF pathway either by the direct inhibition of VEGF (e.g., Bevacizumab) or by the inhibition of autophosphorylation of VEGFR2 (e.g., sunitinib, sorafenib) has become a therapeutic target for cancer therapy and other angiogenesis-dependent diseases [25,26,27]. This approach has been shown to contract the tumor size and prevent its further growth. Therefore, several angiogenesis inhibitors (antiangiogenic agents) have entered clinical practice [28,29,30,31,32]. 

A plethora of evidence for cancer treatment indicate the usage of a single anticancer drug such as doxorubicin (Dox), paclitaxel (PTX), camptothecin (CPT), docetaxel (DTX), cisplatin, 5-and fluorouracil, etc. [33,34,35,36,37,38]. However, the main limitations of these compounds are the lack of selectivity and dose-dependent side effects, such as bone marrow toxicity, cardiotoxicity, nephrotoxicity, and hepatotoxicity [39,40]. To overcome these limitations, in the past few decades numerous nanoformulations—including liposomes, polymer-drug conjugates, polymeric nanoparticles, micelles, hydrogels, mesoporous silica nanoparticles (MSNs) and so forth—of these anticancer drugs have been extensively investigated. Some of the advantages of these drug delivery systems are high efficacy, increased tumor selectivity, reduced side effects, and the imparted enhanced water solubility of the carried drug [41,42,43,44,45]. The high efficacy and increased tumor selectivity are due to their prolonged circulation time and selective accumulation into tumor tissue through the enhanced permeability and retention (EPR) effect [46,47,48,49]. These nanosized drug delivery systems have been found to be promising in the field of cancer therapy due to their improved pharmacokinetic (PK) profiles over low molecular weight drugs [50,51,52]. The clinical success of these nanoformulations has already been proven with marketed products—e.g., Doxil^®^, a liposomal formulation of Dox. This was the first nanoformulation that has been successfully approved by the Food and Drug Administration (FDA) for the treatment of Kaposi’s sarcoma and other cancers [53]. Several other FDA-approved nanoformulations (e.g., Abraxane^®^, DaunoXome^®^) mainly reduce toxicity of the parent compound and thereby improve its therapeutic index [54,55]. Similarly, another nanoformulation—e.g., the so-called polymer-drug conjugate (PDC) of PTX—has been synthesized, and showed a reduced toxicity in comparison to free PTX; it has now reached the advanced clinical trials phase (Phase III) [56,57,58,59].

The clinical success of the aforementioned nanoformulations used for cancer treatment may not be sufficient for therapeutic efficacy, which may be attributed to the physiological complexity of the tumor microenvironment. Combination chemotherapy is widely exploited to achieve the best therapeutic effect from bench to bedside [60,61,62,63,64,65,66,67,68,69,70,71,72,73,74,75]. Cancer combination therapy is usually designed to achieve a therapeutic synergy that is greater than the sum of each drug treatment alone. Following this, the FDA approved a liposome-based nanoformulation of CPX-351 (Vyxeos^®^) for the treatment of acute myeloid leukemia, which delivered two of the most commonly used anticancer drugs (cytarabine and daunorubicin with a fixed 5:1 molar ratio) [76,77,78]. Another liposome-based nanoformulation of a combination of two anticancer drugs, irinotecan and floxuridine, was also reported for the treatment of advanced colorectal cancer that has now reached in phase II trials [79].

**Figure 1:**
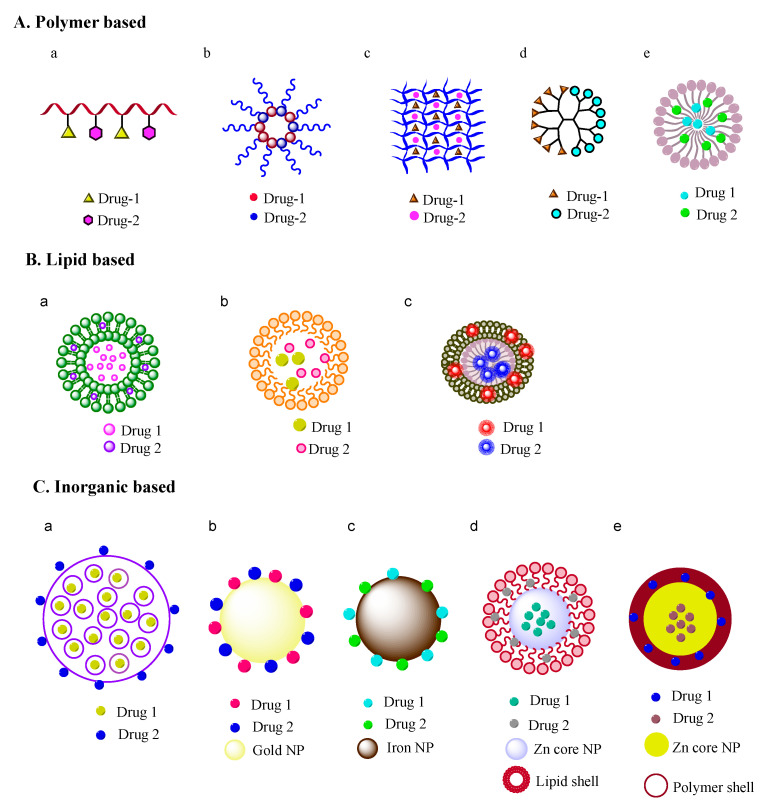
Nanoformulations for combination therapy. (**A**) polymer based nanoformulations: (a) polymer-drug conjugate, (b) micelle, (c) hydrogel, (d) dendrimer, (e) polymeric nanoparticle; (**B**) Lipid based nanoformulations: (a) liposome, (b) solid lipid nanoparticle, (c) nanocell; (**C**) Inorganic material based nanoformulations: (a) mesoporous silica particle, (b) gold nanoparticle, (c) iron nanoparticle, (d) lipid-core/shell nanoparticle, (e) polymer-core/shell nanoparticle. (NP denotes nanoparticle).

**Figure 2:**
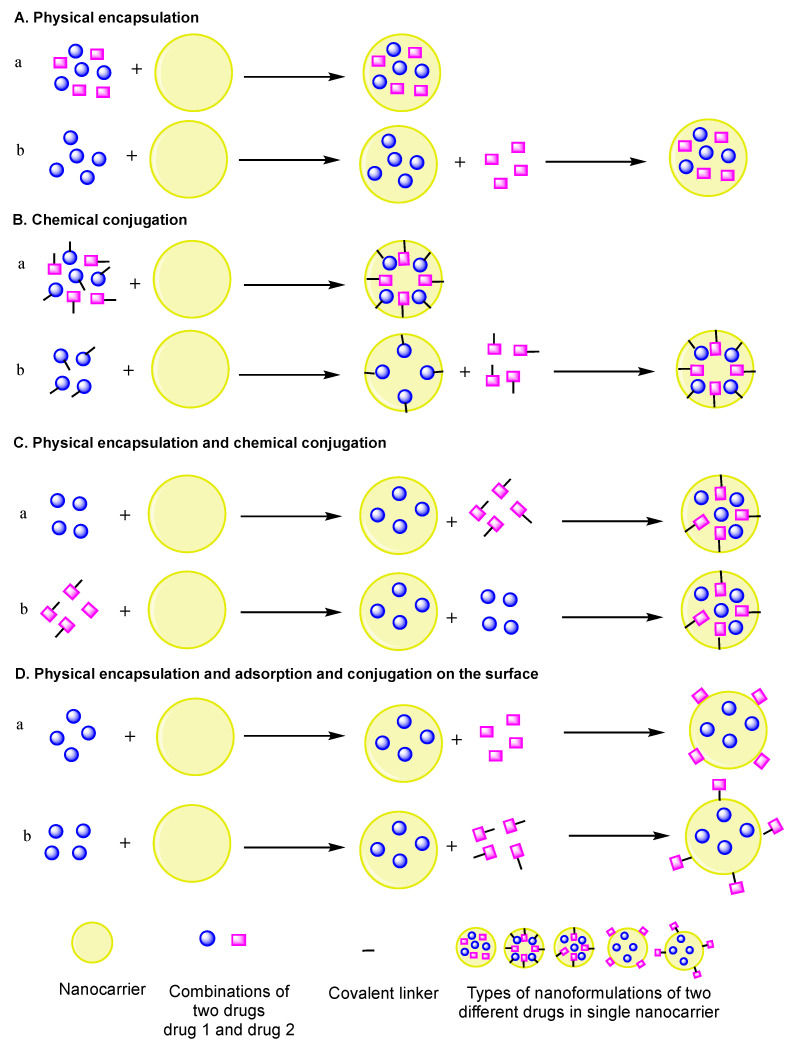
Pictorial representation of different approaches to implement two drugs in a single nanocarrier. (**A**) Physical encapsulation: (a) two different drugs physically encapsulated, (b) two different drugs physically encapsulated one after the other; (**B**) chemical conjugation: (a) two different drugs chemically conjugated, (b) two different drugs chemically conjugated one after the other; (**C**) physical and chemical conjugation of two different drugs one after the other: (a) drug1 physically encapsulated and drug2 chemically conjugated, (b) drug1 chemically conjugated and drug2 physically encapsulated; (**D**) physical encapsulation and adsorption and conjugation on the surface: (a) drug1 physically encapsulated and drug2 absorbed on the surface, (b) drug1 physically encapsulated and drug2 chemically conjugated on the surface.

Numerous other nanoformulations of combinations with two different mechanisms of action of the drugs (e.g., anticancer/antiangiogenic drugs) in optimized ratios have also been explored. However, such systems have not been extensively clinically explored, despite their exploration in preclinical studies. The importance of combining conventional chemotherapy with antiangiogenesis can be explained through tumor vascularization, as both the tumor growth and metastasis are dependent on it. Hence, the combination therapy with these two agents can prevent the tumor recurrence and improve the antitumor effect. Nanoformulations of anticancer/antiangiogenic drugs would allow the optimization of drugs to reduce the dose-related side effects and maximize the synergy of the drugs. Recently developed nanoformulations of anticancer and antiangiogenic drugs in a nanocarrier using different approaches covered in this review are presented in Figure 1 and Table 1. Similarly, different approaches for the implementation of two drugs in a nanocarrier are depicted in Figure 2. Hence, this review explores ongoing research and presents future prospects for the potential therapeutics of nanoformulations of anticancer and antiangiogenic drug combinations for more effective combination cancer therapy.

## 2. Nanoformulations of Anticancer and Antiangiogenesis Drugs for Combination Cancer Therapy

Organic (e.g., polymers, lipids) and inorganic (e.g., mesoporous silica nanoparticles or MSNs, gold) material-based nanoformulations have been extensively developed, investigated, and used for dual-drug combinations. In particular, nanoformulations presented in Table 1, developed to deliver antiangiogenic and anticancer drugs in order to increase their therapeutic efficacies, are discussed below for their use in combination cancer therapy. The combination of the drugs used in the below-mentioned nanoformulations have been reported to act in a synergistic manner, where the cumulative effect of the antiangiogenic and anticancer drugs is greater than the sum of the individual effects of each drug. 

### 2.1. Polymeric-Based Nanoformulations

Polymers offer superior advantages and vast usage for nanodrug delivery among all the commonly used biodegradable nanomaterials. Biocompatible polymers provide a versatile platform to load multiple low molecular weight drugs to generate different types of nanoformulations, such as polymer nanoparticles, polymer-drug conjugates, polymer-based micelles, dendrimers, and hydrogels. Hence, this section highlights and discusses the recent advances and impacts of polymer-based nanoformulations, which have shown potential effectiveness for combination cancer therapy.

**Figure 3:**
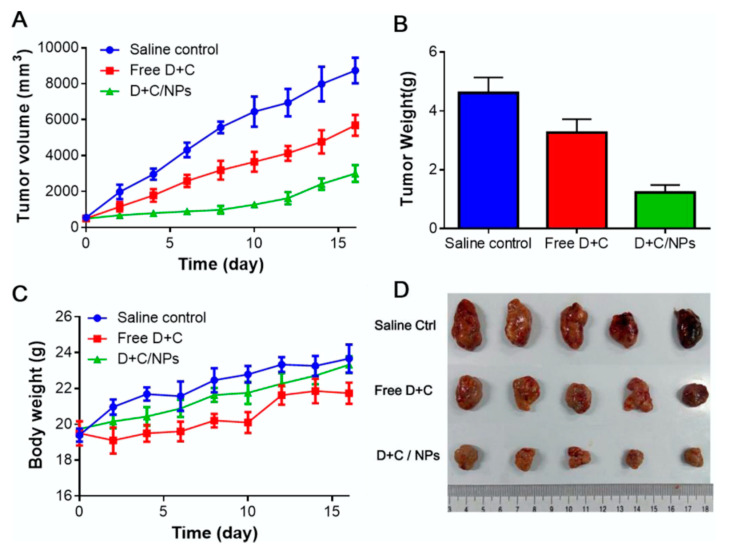
Antitumor effect of pH-sensitive polymeric nanoparticles of co-loaded Dox and Cur in hepatocellular carcinoma: (**A**) Tumor volume, (**B**) tumor weight, (**C**) body weight at the end of the experiment, (**D**) picture of excised tumors at the end of the experiment [80]. Reprinted with permission from ref. [80], copyright (2017) Elsevier.

Jinming Zhang et al. developed a pH-sensitive polymeric nanoparticle using an amphiphilic poly (*b-*amino ester) copolymer to deliver Dox and curcumin (Cur) in optimized ratios for the treatment of hepatocellular carcinoma (HCC) [80]. Dox is commonly used as anticancer drug that causes DNA damage and activate apoptosis. Cur is a bioactive compound derived from the herb Curcuma longa L. (known as turmeric) and exhibits a potent antiangiogenic activity. The Dox and Cur nanoformulation showed a low polydispersity, high encapsulation efficiency, and enhanced release in the acidic environment of tumor cells. Likewise, an enhanced cellular internalization was observed in human liver cancer cells and human umbilical vein endothelial cells as compared to their free-drug counterparts. This nanoformulation also exhibited a high rate of apoptosis in human liver cancer cells and greater antiangiogenic effects both *in vitro* and *in vivo* (Figure 3). Overall, this pH-sensitive polymeric nanocarrier containing Dox and Cur drugs was demonstrated to inhibit cancer cell proliferation and angiogenesis in a synergistic manner, suppressing the tumor growth in HCC [80]. 

**Figure 4:**
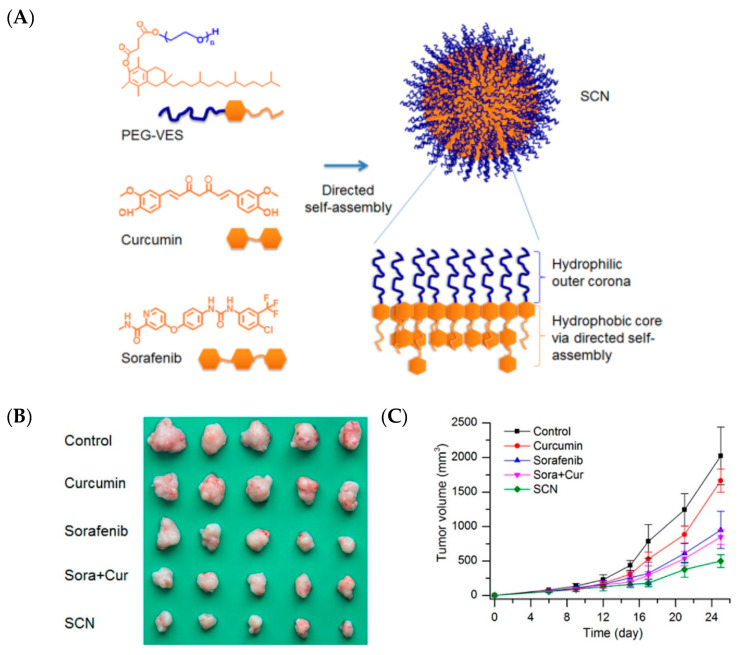
Self-assembled nanoparticles (SCNs) for the co-delivery of sorafenib and curcumin enhance their therapeutic effect in hepatocellular carcinoma: (**A**) schematic representation of SCNs, (**B**) tumor tissues from each group, (**C)** tumor growth profile [81]. Reprinted with permission from ref. [81], copyright (2015) American Chemical Society.

In another study, Haiqiang Cao et al. designed polymeric self-assembled nanoparticles (SCNs) with sizes of 84.97 ± 6.03 nm (homogeneous nanometric spherical particles) to enhance the therapeutic effect in HCC. This nanoparticle comprised of two hydrophobic drugs-sorafenib (Sora) and Cur- and polyethylene glycol-vitamin E succinate (PEG-VES). These could be directly self-assembled into SCN due to their intermolecular hydrophobic interactions, thereby combining two drugs within a single nanocarrier [81]. These SCNs presented superior effects *in vitro* (using BEL-7402 and Hep G2 cancer cells) in comparison to Sora, Cur, and their physical mixture (Sora + Cur) in terms of enhanced cytotoxicity, cell apoptosis, and antiangiogenesis activities in tube formation and micro vessel formation from aortic rings. Specifically, in a tumor xenograft model of human hepatocellular carcinoma cell lines of BEL-7402, SCN showed an enhanced inhibitory effect on tumor progression when compared to free drugs or their physical mixture, along with significantly greater antiproliferation and antiangiogenesis properties. Thus, these *co-*delivered nanoassemblies of Sora and Cur have been shown to enhance the therapeutic effect on antiangiogenesis and antiproliferation activities for combination cancer therapy in an *in vivo* model of hepatic cellular carcinoma (Figure 4) [81].

**Figure 5:**
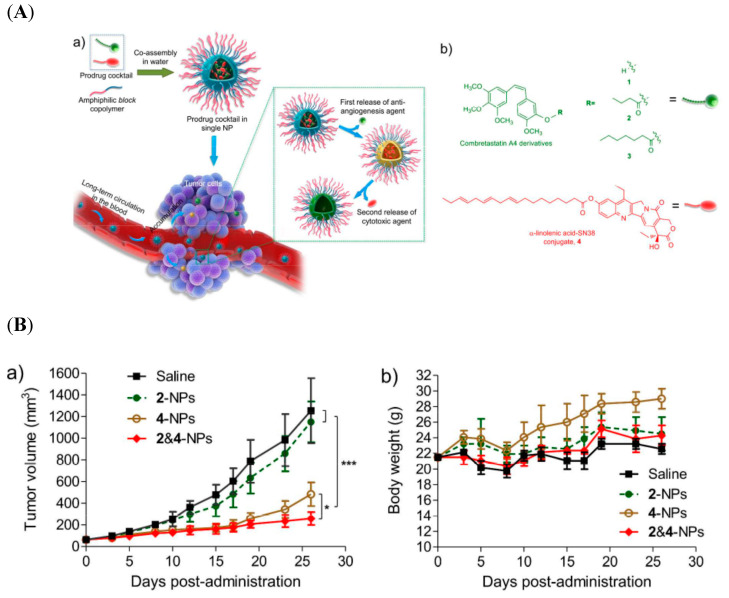
(**A**) (a) Schematic illustration of self-assembled nanoparticles of block copolymers and prodrugs of CA4 derivatives (2 and 3) and linolenic acid-7-ethyl- 10-hydroxycamptothecin (SN38) conjugates; (b) chemical structures of CA4 and its ester derivatives 2 and 3 and derivatives of SN38. (**B**) Mice bearing subcutaneous HT-29 tumors were intravenously treated with different drug-loaded nanoformulations three successive times: (a) change in tumor, (b), body weight change [82]. Reprinted with permission from ref. [82], copyright (2017) American Chemical Society. * *p* < 0.05, *** *p* < 0.001.

Prodrug-assembled polymeric nanoparticles for the delivery of two drugs [CA4 and 7-ethyl- 10-hydroxycamptothecin (SN38)] in a sequential manner on demand at the target site was developed by Hangxiang Wang et al. CA4, an antiangiogenic agent, can disrupt the tumor neovasculature, causing vascular shutdown, while SN38, a chemotherapeutic drug, can inhibit the DNA topoisomerase 1 of cancer cells. The obtained amphiphilic-assembled polymeric nanoparticles exhibited a sequential release of two drugs: initially, CA4, followed by SN38. A HT-29 colon tumor-bearing mousse model in an *in vivo* study indicated that the CA4/SN38 *co-*encapsulated polymeric nanoparticles displayed synergistic activities in inhibiting the tumor growth (Figure 5) [82].

**Figure 6:**
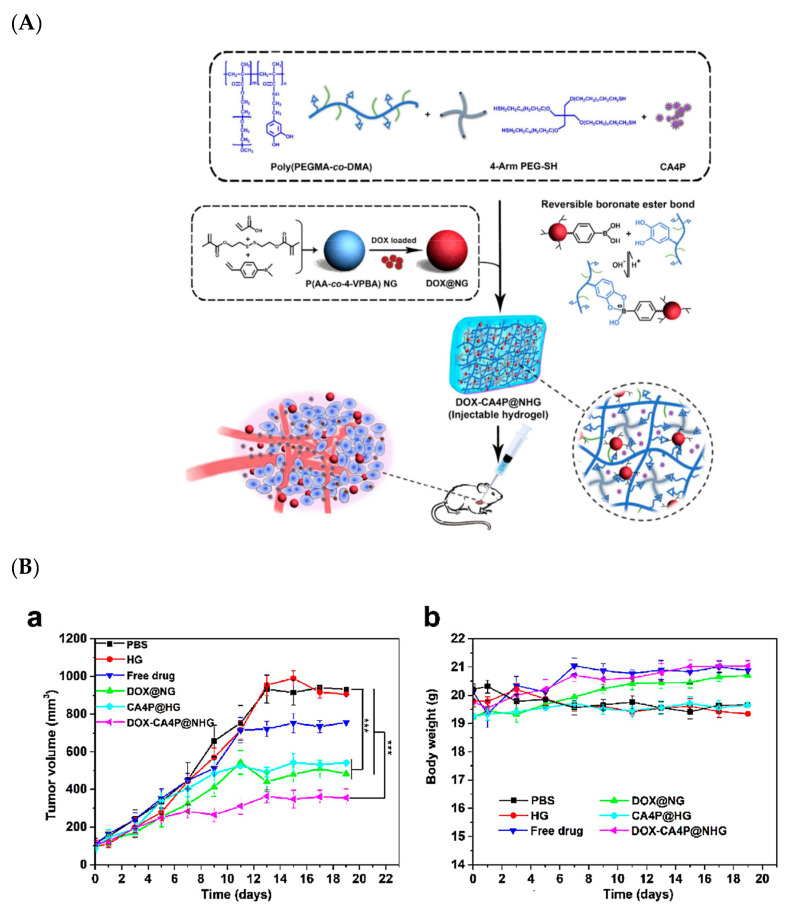
(**A**) Chemical scheme and schematic representation of nanohydrogel for the sequential local delivery of CA4P and Dox, (**B**) (a) tumor volumes in tumor regression study of hepatocellular carcinoma (HCC) (tumor-bearing mice), (b) tumor body weight changes [83]. Reprinted with permission from ref. [83], copyright (2018) American Chemical Society. *** *p* < 0.001.

Wen Jing Yang et al. attempted using poly(acrylic acid*-co-*4-vinylphenylboronic acid) to develop a synergistic pH/redox stimuli-responsive nanohydrogel for the sequential local delivery of combretastatin A-4 phosphate (CA4P) and Dox for antiangiogenesis and anticancer combination therapy. This nanoformulation also released CA4P and Dox in a sequential manner at the target site on demand. A cumulative release was observed, with about 57.2% released at 9 h and almost 90.7% after 48 h at pH 6.5. This nanogel exhibited a high inhibitory activity on the cancer cell proliferation (MCF-7 and normal 3T3-L1 cells) *in vitro*, with a superior antitumor therapeutic efficacy with a single injection in HCC xenograft tumor-bearing mice (Figure 6) [83].

Mohyeddin Assali et al. prepared self-assembled micelles combining combretastatin A4 (CA4) and camptothecin (CPT) by using click chemistry. These micelles displayed enhanced stability and water solubility at pH 7.4, with a low critical micelle concentration (CMC) of 0.9 mM. Furthermore, this micelle formulation encompassing two drugs displayed five times higher cytotoxicity against HeLa cancer cells when compared to the free drugs. Moreover, a combination index (CI) of less than 1 suggested a synergistic activity by the micelles. Imaging studies of HeLa cells treated with FITC-loaded micelles showed a rapid internalization. Based on these results, *in vivo* studies to determine the anticancer activity were suggested [84].

**Figure 7:**
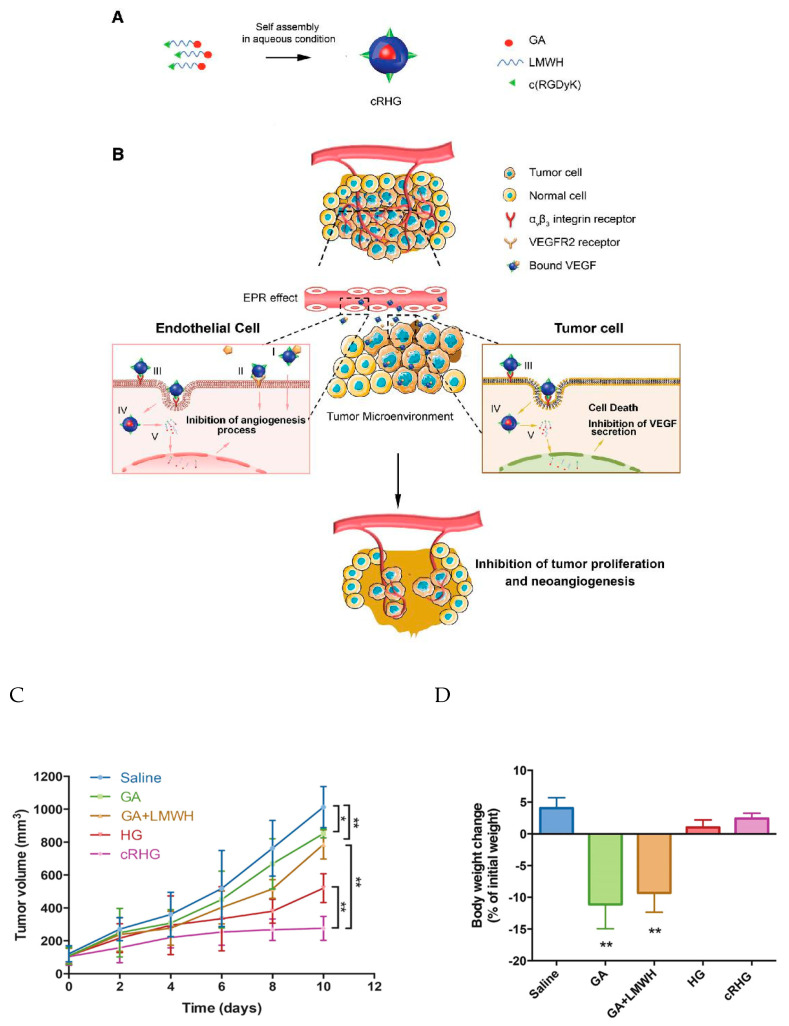
(**A**) Schematic representation of the self-assembled heparin–gambogic acid conjugate (cRHG) nanoparticles; (**B**) passive accumulation of cRHG in the tumor site via the enhanced permeability and retention (EPR) effect targeted at tumor cells and neovasculature. (I) Binding to angiogenic factors (VEGF), (II) inhibition of VEGFR2 phosphorylation, (III) recognition of cRHG by αvβ3 integrin, (IV) αvβ3 integrin-mediated internalization of cRHG, (V) disassembly and drug release. (**C**) *In vivo* antitumor efficacy assay-the U87MG tumor growth curves after the intravenous injection of different GA formulations at a dose of 2 mg kg^−1^; (**D**) body weight changes of U87MG tumor-bearing mice after 10 d administration of different formulations [85]. Reprinted with permission from ref. [85], Copyright (2016) John Wiley & Sons, Inc. * *p* < 0.05 and ** *p* < 0.01.

Fatima Zohra Dahmani et al. developed a heparin–gambogic acid conjugate (cRHG) and c(RGDyK)-functionalized (targeting ligand) self-assembled polymeric amphiphilic nanoformulations. These nanoformulations showed considerable the inhibition of VEGF, hypoxia inducible factor-1 alpha, and CD31 expression, with the significant downregulation of phosphorylated vascular endothelial growth factor receptor-2 (pVEGFR2). These results also demonstrated a versatile nanoplatform for efficient combinational tumor therapy *in vivo* (Figure 7). Hence, a combination of chemo- and antiangiogenesis therapy holds immense potential for effective tumor growth inhibition [85]. 

**Figure 8:**
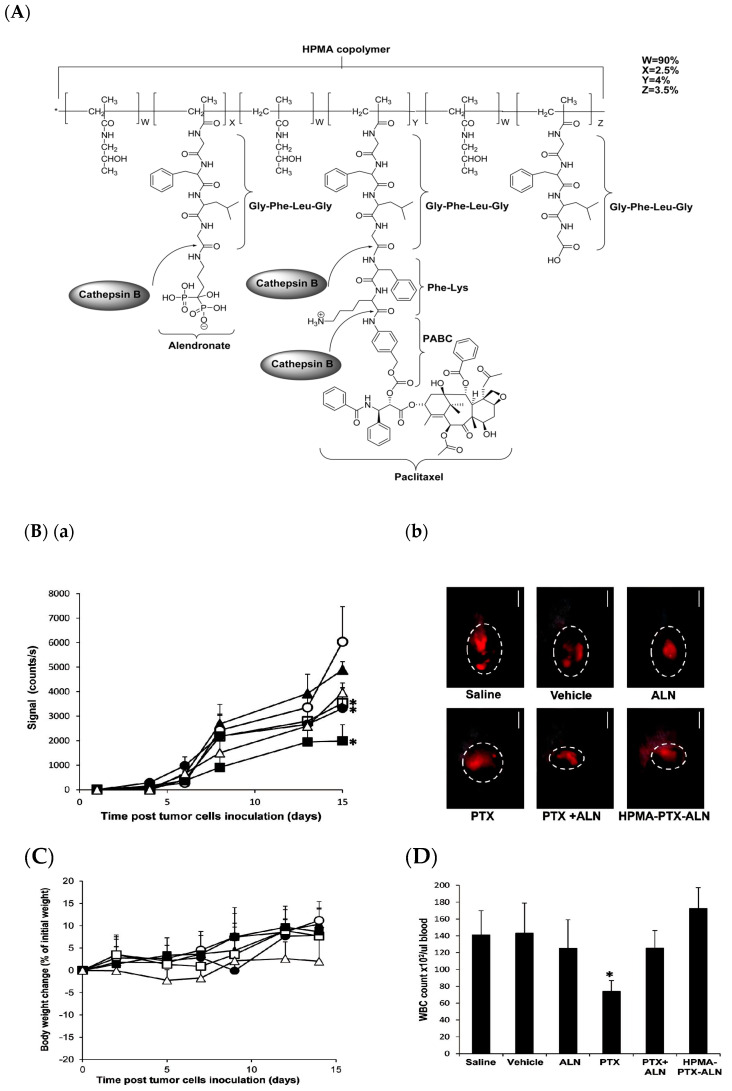
(**A**) Chemical structure of *N*-(2-Hydroxypropyl)methacrylamide (HPMA) copolymer- paclitaxel (PTX)-alendronate (ALN) conjugate and the release of the drugs PTX and ALN by cathepsin B; (**B**) conjugate inhibits 4T1-mCherry mammary adenocarcinoma in the tibia, (**a**) antitumor efficacy measured by intravital noninvasive fluorescence imaging, (b) fluorescence images; (**C**) body weight change (presented as % change from initial weight); (**D**) White blood cells (WBC) counts from blood samples collected on day 11. Data represent mean (SEM of six mice per group. * *p* < 0.05 value of mice treated with HPMA copolymer-PTX-ALN conjugate was analyzed against saline control mice. * *p* < 0.05 value of free PTX or combination of free PTX plus ALN was analyzed against control mice treated with PTX vehicle. [86]. Reprinted with permission from ref. [86], copyright (2011) American Chemical Society.

Keren Miller et al. engineered a novel polymer-drug conjugate, namely *N*-(2-Hydroxypropyl)methacrylamide (HPMA)-copolymer-paclitaxel-alendronate (HPMA-PTX-ALN) conjugate, to improve antitumor and antiangiogenic activity in breast cancer. The chemical synthesis scheme (Figure 8A) shows that PTX and ALN were conjugated to HPMA co-polymer via a phe-lys-p-aminobenzyl carbonate (FK-PABC), which showed a sustained release by cathepsin B. This conjugate showed the highest antitumor activity compared to free PTX or with a combination of free PTX plus ALN in 4T1-mcherry mammary adenocarcinoma in the tibia (Figure 8) [86].

**Figure 9:**
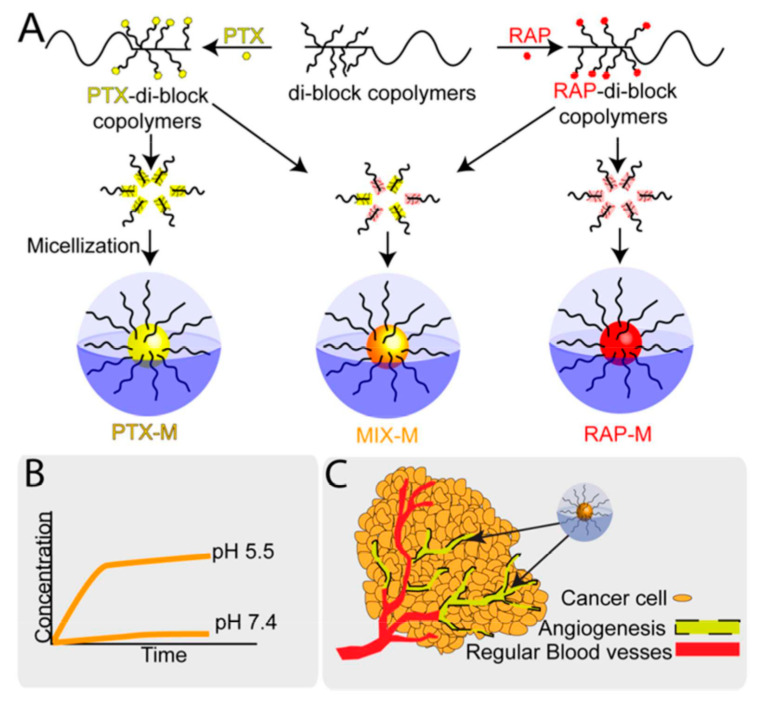
Pictorial representation of individual and mixed micelle polymer-conjugates of paclitaxel (PTX) and rapamycin (RAP): (**A**) synthesis schematic for polymeric-paclitaxel conjugate and polymeric-rapamycin conjugate and their micelles formulation, (**B**) pH-dependent drug release from micelles, (**C**) accessibility and accumulation of micelles for human umbilical vein endothelial cells HUVECs and cancer cells (ovarian) in the tumor environment [87]. Reprinted with permission from ref. [87], copyright (2016) American Chemical Society.

Deepa A Rao et al. developed individual and mixed micelles of polymeric-paclitaxel (PTX) conjugates and polymeric-rapamycin (RAP) conjugates with an acid-sensitive linker. This acid-sensitive linker released the drugs from the micelles in a preferential manner at pH 5.5 in lysosomes. The micelles displayed antiproliferative activity in a synergistic manner against ovarian cell lines (human Caucasian ovarian adenocarcinoma and human ovarian clear cell carcinoma) and endothelial cell line. The inhibition of endothelial cell migration and tube formation were also reported. No acute toxicity was observed in healthy mice for over 21 days at a dose of 60 mg/kg of the micelles. The individual micelles exhibited only antiangiogenic activity, whereas the mixed micelles demonstrated both antiangiogenic and apoptosis induction activity in an epithelial ovarian cancer xenograft model in efficacy studies at 20 mg/kg/drug dosed every 4 days and assessed after 21 days (Figure 9) [87]. 

**Figure 10:**
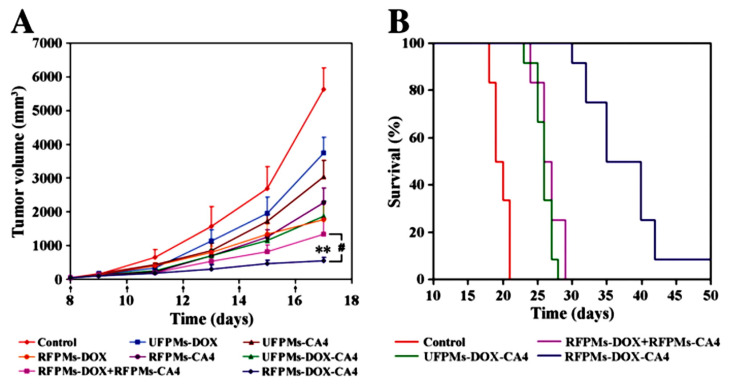
Antitumor activity and systemic toxicity of arginine-glycine-aspartic acid (RGD)-functionalized polymeric micelles of Dox-CA4 micelles in melanoma B16-F10 tumor-bearing mice: (**A**) tumor volumes of different treatment groups, (**B**) survival of tumor-bearing mice [88]. Reprinted with permission from ref. [88], copyright (2011) Elsevier. ** *p* < 0.01.

Yiguang Wang et al. synthesized arginine-glycine-aspartic acid (RGD)-functionalized polymeric micelles of poly(ethylene glycol)-*b*-poly(d,l-lactide) (PEG-PLA). Here, Dox and CA4 were encapsulated into micelles (namely, RFPMs-DOX-CA4) of 29.2 ± 2.5 nm size. These micelles (RFPMs-DOX-CA4) was demonstrated to sequentially release both drugs, resulting in the sequential killing of endothelial cells and tumor cells *in vitro*. In addition, these micelles were reported to exhibit a greater tumor growth inhibition and higher survival rate in comparison to free drugs and their combination in B16-F10 tumor-bearing mice. Further, the *in vivo* evaluation of these micelles showed a significant reduction in the tumor vasculature and cell proliferation, which suggests these micelles can be effective for combination cancer therapy (Figure 10) [88].

In summary, this section explored cancer targeting through polymer-based nanoformulations of anticancer and antiangiogenic agents in a single nanocarrier for combination therapy at lower doses, with an aim to reduce the toxicity and enhance the therapeutic efficacy.

The hallmark of these above-mentioned nanoformulations is the selection of appropriate polymers, which could be based on polymeric nanoparticles, polymeric conjugates, micelles, and hydrogels. The ideal polymer would be one which is non-toxic, water soluble, non-immunogenic, and has a high drug loading capacity. Apart from this, one of the key factors that plays a critical role in polymer pharmacokinetics and biodistribution is the molecular weight (MW) of the polymer, which ensures a long circulation in the bloodstream to allow EPR-mediated accumulation. Indeed, for non-biodegradable polymers, the MW must be less than 40–50 kDa (renal clearance threshold) to ensure renal elimination [95]. In general, a MW of 30–100 kDa is employed as an optimum range for drug delivery; however, it needs to be tailored to the particular polymer based on its architecture and biodegradability. Considering the key attributes of the polymers, as stated above, significant efforts were made to elucidate the synergetic effects achieved by the combination therapy, which led to advanced drug delivery strategies rather than simply an additive effect of the partner drugs [96,97].

For instance, *D*-a-tocopheryl poly-ethylene glycol-block-poly (*b-*amino ester) amphiphilic copolymers used a pH-sensitive nanoparticle with an enhanced pH sensitivity and stability in the physiological environment and encapsulated anticancer and antiangiogenic drugs simultaneously by self-assembly [80]. In another study, a PEG derivative of vitamin E succinate and CA4 and SN38 derivatives could be directly self-assembled into polymeric nanoparticles due to the intermolecular hydrophobic interactions among them, which combined two drugs within a nanovehicle to exert the desired effect for effective combination therapy [81]. In another experiment, polymer-based hydrogel was used to deliver antiangiogenic and anticancer drugs with sequential drug release profiles, which subsequently led to the sustained long-term release of the drugs [83]. In one study, HPMA copolymer facilitated the attachment of chemotherapeutic and antiangiogenic drugs to a polymeric backbone, as well as targeting moieties, such as the bone-targeting agent ALN [86]. Overall, these polymer-based nanoformulations help with implementing a robust and successful combinational cancer therapy.

### 2.2. Lipid Based Nanoformulations

Lipid-based nanocarriers, such as liposomes, solid lipid nanoparticles, nanocells, and lipid-coated nanoparticles have emerged as promising nanovehicles for cancer therapy. This section focusses on the recent advancements of lipid-based and other organic nanoformulations for combination chemo- and antiangiogenesis therapy.

Shiladitya Sengupta et al. incorporated two drugs (Dox and CA4) for a lipid-based nanoformulation in a two-step process. CA4 was incorporated into the lipid layer, whereas Dox was loaded into the polymeric core. The nanoformulation was preferentially taken up by the tumor within 5 h and inhibited the temporal targeting of tumor cells and neovasculature together in a sequential manner and retained for at least 24 h. The synergistic effects of inhibiting the tumor vessels and proliferation of tumor cells were achieved in *in vitro* and *in vivo* studies [89].

In another study, Wenbing Dai et al. constructed a novel liposomal delivery system with the traditional chemotherapy drug Dox and antiangiogenesis agent CA4 with surface modification by the targeting ligand octreotide (Oct). The release kinetics of drugs from the nanoformulation confirmed the rapid release of CA4, followed by a slow release of Dox in vitro. In addition, sequential killing effects were confirmed *in vivo* using nude mice bearing MCF-7 tumors. The active targeted liposomes Oct-liposome of CA4 and Dox showed a specific cellular uptake through ligand-receptor interaction and a higher antitumor effect *in vitro* against somatostatin receptor (SSTR) positive cell lines [98,99,100]. This study concluded that the liposome-based nanoformulation can be a potential nanodrug delivery system for the treatment of malignant solid tumors [90].

**Figure 11:**
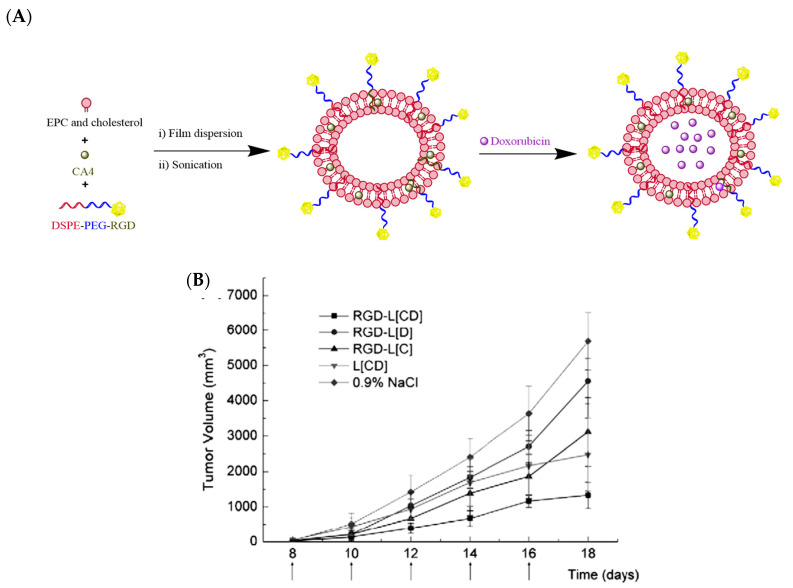
(**A**) Pictorial representation of arginine–glycine–aspartic acid (RGD)-modified liposomes loaded with Dox and CA4 (RGD-L-CA4/Dox); (**B**) tumor volume graph showing the tumor growth inhibition of RGD-L-CA4/Dox in comparison to single drug-loaded liposomes at the doses of 25 mg CA-4/kg and 0.8 mg Dox/kg, with saline as a control in C57BL/6 mice bearing B16F10 tumors [91]. Reprinted with permission from ref. [91], copyright (2010) Elsevier.

Yi-Fei Zhang et al. developed an arginine–glycine–aspartic acid (RGD)-modified liposome, co-encapsulating Dox and CA4 with the aim of assessing sequential release and enhancing tumor inhibition responses. The results showed that the release rate of Dox was much slower than that of CA4 *in vitro*. The intracellular uptake of liposomal drugs by B16/B16F10 melanoma tumor cells and human umbilical vein endothelial cells (HUVECs) was enhanced based on flow cytometry and laser confocal scanning microscopy assessments. A cytotoxicity assay demonstrated the lower half maximal inhibitory concentration (IC_50_) of RGD-modified liposomes than the corresponding unmodified liposomes. Prominent synergistic effects on tumor reduction were observed with RGD-modified liposomes co-encapsulating CA4 and Dox, delineating the importance of a targeted drug delivery system for the co-encapsulation of antiangiogenic and anticancer agents for cancer treatment (Figure 11) [91].

In summary, the development of lipid-based nanoparticles/nanocarriers, and particularly liposomes, as discussed in the above-cited studies, has been reported as an alternative modality for cancer therapy. These lipid-based nanoformulations work by means of passive and active targeting, thereby reducing the toxicity associated with anticancer and antiangiogenic agents and subsequently improving the efficacy of these drugs similarly to various other types of nanoformulations. Nonetheless, lipid-based nanoparticles have been found to be more beneficial than some of their counterparts, owing to their ingredients being more biocompatible and biodegradable in nature. Moreover, the amphiphilic properties of liposomes allow these nanoparticles to encapsulate both hydrophobic and hydrophilic anticancer and antiangiogenic drugs. Hence, lipid-based nanoparticles may help in improving the therapeutic efficacy and safety profile of combination chemotherapy and, ultimately, the prognosis of cancer patients.

### 2.3. Inorganic Material-Based Nanoformulations

As evident from the above-mentioned numerous nanoformulations, most nanodrug delivery systems in clinical trials and in clinical use are based on organic (mostly liposomal or polymer) platforms. In particular, liposomal formulations- e.g., Doxil—used for monotherapy for cancer as well in combination chemotherapy have been successfully translated to the clinic. Meanwhile, nanoformulations based on inorganic materials such as MSNs, iron oxides (Fe_3_O_4_), gold (Au), and silver (Ag) have also become a very important class of nanodrug delivery vehicle. The following discussion illustrates the recent advances in inorganic-based nanoformulations, especially MSNs, iron oxide, and gold-based nanoformulations, for chemo and antiangiogenesis combination cancer therapy.

**Figure 12:**
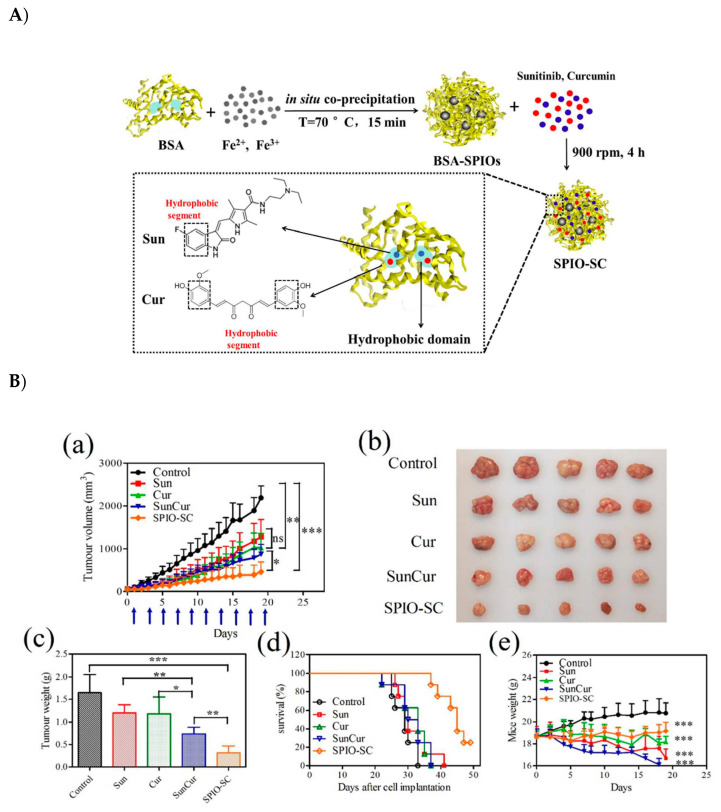
(**A**) Schematic representation of bovine serum albumin-coated superparamagnetic iron oxide (BSA-SPIO) nanoparticles further co-loaded with two drugs (Sun and Cur) to achieve combined cancer therapy. (**B**) antitumor activity of BSA-SPIOs and drug-loaded BSA-SPIOs in an MCF-7 tumor xenograft mouse model. The mice were treated with free Sun, free Cur, free Sun + Cur, and SPIO-SC every other day by tail vein injection at doses of 15 mg/kg for sun and 30 mg/kg for cur. (a) The growth of tumor volumes, (d) Nude mouse survival time of each group; (e) Nude mouse weight of each group [92]. Reprinted with permission from ref. [92] copyright (2017) Royal Society of Chemistry. * *p* < 0.05, ** *p* < 0.01, *** *p* < 0.001.

Sunhui Chen et al. developed bovine serum albumin-coated superparamagnetic iron oxide (BSA-SPIO) nanoparticles and further *co-*loaded this nanosystem into two drugs—the cytotoxic drug Cur and tyrosine kinase inhibitor (TKI) sunitinib (Sun)—to achieve synergistic effect. The BSA-SPIOs and dual-drug (Sun and Cur)-loaded BSA-SPIO nanoparticles, when compared to free drugs, displayed the most significant tumor inhibition in an MCF-7 tumor xenograft mouse model. In addition, these NPs were used for *in vivo* MR imaging and prompted a good targeting to the tumor site with well-preserved stability and long-circulation potential, rendering them promising candidates for both tumor diagnosis and therapy. These nanoparticles exhibit both *in vivo* MR imaging and tumor therapy capability, thus being a promising theragnostic material (Figure 12) [92].

**Figure 13:**
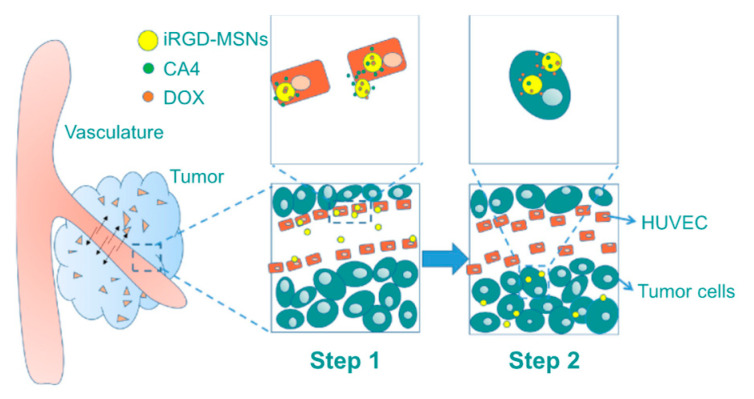
Pictorial representation of CA4 and Dox-loaded mesoporous silica nanoparticles (MSNs) targeted with 9-amino acid (CRGDKGPDC) cyclic (iRGD) peptide delivery in tumors. Step 1: CA4 is first released at tumor vasculature with the help of targeting ligands (iRGD peptides); subsequently, in step 2 this nanoformulation is endocytosed into acidic tumor cells where most of the Dox is released [93]. Reprinted with permission from ref. [93], copyright (2016) Dove press.

Xiaoyu Li et al. reported a MSN-based nanoformulation loaded with two different drugs, CA4 and Dox. Additionally, 9-amino acid (CRGDKGPDC) cyclic (iRGD) peptide was used as a targeting ligand conjugated onto the MSN surface. Particularly, iRGD peptide targeted α_2_β_3_ integrin receptors, which are overexpressed in cancer and tumor vascular cells. Therefore, this nanocarrier targeted tumor vasculature specifically and was used for combined chemo- and antiangiogenesis therapy. When these dual-drug-loaded MSNs were injected into the blood circulation, they accumulated at the targeted tumor via the α_2_ β_3_ integrin receptors in the tumor environment. Most of the antiangiogenic drug was released first, while only a small amount of Dox was released during the same time period due to the negatively charged MSNs interacting electrostatically and via hydrogen bonding with the positively charged Dox. Sequentially, after reaching deep into the tumor, Dox quickly released in the lower pH environment. The further uptake by tumor cells and release of Dox efficiently induced the apoptosis of the cancer cells. Such nanoformulation showed a synergetic effect and greatly enhanced the cytotoxic effect of Dox in cancer cells (Figure 13) [93].

**Figure 14:**
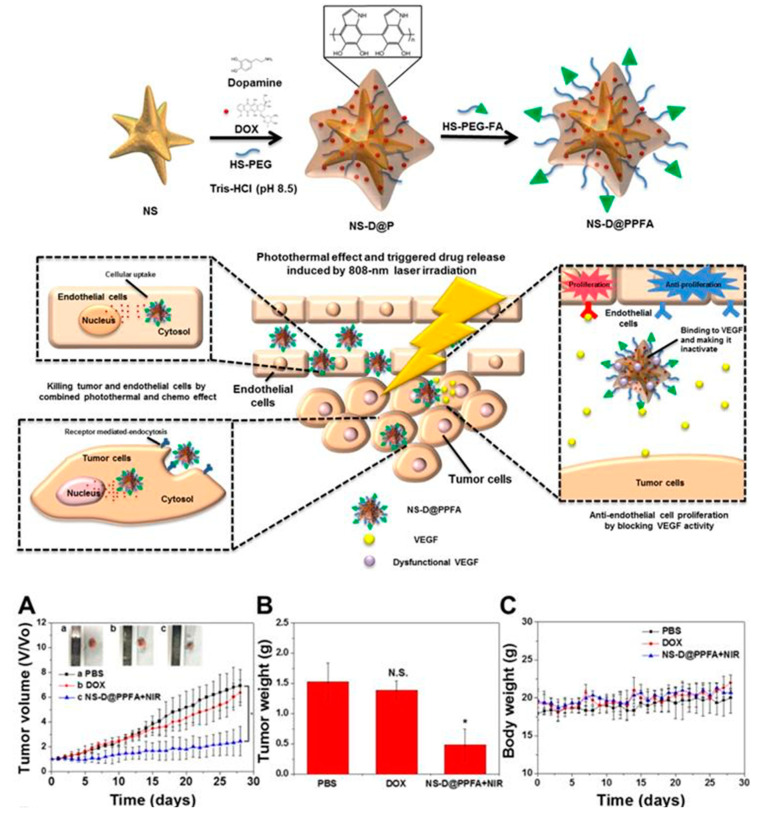
Schematic representation of the synthesis of polydopamine (PDA)-coated gold (Au) nanostar (NS@PPFA) for combined anticancer and antiangiogenic treatment: antitumor activity of polydopamine (PDA)-coated gold (Au)-Dox nanostar (NS-D@PPFA) in MCF-7/Adriamycin (ADR) tumor-bearing mice. (A) Tumor growth curve of mice receiving intravenously administration of PBS, Dox (5 mg/kg), and NS-D@PPFA (6 mg/kg Au; 1.8 mg/kg Dox) at day 0 and day 14, respectively. Tumors were treated with NIR irradiation (0.9 W/cm^2^, 3 min, 3 times) at 24 h and 48 h after each injection. (B) Tumor weight was measured on excised tumors at day 28 after different treatments. (C) Body weight monitoring of the treated mice over a period of 28 days [94]. Reprinted with permission from ref. [94]. * *p* < 0.05, N.S.: not significant.

You-Hong You et al. engineered a multifunctional (PDA)-coated gold (Au) nanostar (NS@PPFA) nanoformulation containing Dox (NS-D@PPFA) for combination cancer therapy to target drug resistance in breast cancer. Breast cancer MCF-7 and drug-resistant MCF-7/Adriamycin (ADR) cells demonstrated the effective intracellular uptake and cytotoxicity of the designed nanoformulation. These nanoagents were found to be more active in inhibiting VEGF-induced VEGFR angiogenesis. The CD31 and pVEGFR2 levels were also significantly reduced *in vivo*. The investigation of the antitumor activity of NS-D@PPFA (6 mg/kg Au, 1.8 mg/kg Dox) in comparison to PBS as a control and free Dox (5 mg/kg), plus near-infrared (NIR) laser in MCF-7/ADR tumor-bearing mice, showed that the inhibition of the tumor cells and endothelial cells proliferation was achieved by combined chemo and photothermal effects (chemo-PTT). The photothermal effect and triggered drug release was induced by NIR laser (808 nm)/808-nm irradiation (Figure 14) [94]. From the *in vitro* and *in vivo* results, this nanoformulation simultaneously presented a remarkable antitumor efficacy by chemo-PTT combination therapy, triggered by a single NIR laser (Figure 14) [94]. Overall, this study found to a new therapeutic strategy against antiangiogenic cancer therapy and multidrug-resistant cancers.

In summary, given the variety of inherent properties that different inorganic materials possess—as discussed above, for instance superparamagnetic (iron oxides) and photothermal (gold) activity—these are highly interesting constructs to be included in the design of hybrid nanocarriers for combination therapies. Many inorganic materials possess inherent imaging activity, rendering them suitable to be tracked by different biological or medical imaging techniques. As mentioned above, this not only allows easy tracking in the biological or physiological environment during the investigation of the behavior of the nanocarrier, but also implies their potential as theragnostic agents. A couple of other examples showed how the modularity and robustness of the mesoporous silica matrix could be utilized to load multiple drugs simultaneously, while separately functionalizing the outside particle surface with targeting ligands. Nevertheless, inorganic materials usually require organic functionalization to reach the desired responsiveness and, in many cases, biocompatibility in the physiological environment, as well as for the conjugation of targeting ligands. In the above-discussed cases, organic coatings were utilized, e.g., to facilitate pH-sensitive drug release (PDA) and long circulation time, and low immunogenicity (BSA) and drug loading capability. Such properties are usually dependent on the interaction of the nanosystem with the surrounding environment (sometimes referred to as the “bio-nano interface”), while the inherent properties of inorganic materials (robustness for drug incorporation and protection, imaging activity, photothermal and photodynamic activity) are not in general dependent on direct contact. Consequently, constructing hybrid nanocarriers by making use of an inorganic platform with organic functionalization appears to be an especially flexible approach for the development of synergistic nanocarriers for combined chemo- and antiangiogenetic therapy, even combined with other therapeutic strategies.

## 3. Future Perspectives, Outlook, and Conclusions

On one hand, the drug resistance and multiple side effects associated with conventional chemotherapy remains a significant barrier in the treatment of cancer. On the other hand, monotherapy of antiangiogenic drugs has demonstrated only a temporary response in clinical trials, with some serious dose limiting toxicities and allergic reactions. Nevertheless, the tumor tissue exposure to combination therapy of antiangiogenesis and chemotherapeutic agents with dissimilar chemical and pharmacological properties remains very challenging. To overcome this, various nanoformulations with tunable and predictable release of multiple drugs have been investigated in preclinical studies (mice models), and the same needs to be validated in different species such as rat, rabbit, and monkey and thus eventually among cancer patients before these nanoformulations can be launched into the market.

Despite the wide array of advantages of nanoformulation-mediated combination chemo-antiangiogenesis cancer therapy—e.g., reduction in tumor growth, the regulation of drug resistance, and overall synergistically improved therapeutic efficacy—there are certain challenges that remain in their preparation and efficient translation. These include unknown toxicity and immunogenicity aspects, inefficient drug targeting, the lack of collaboration from bench to bedside between experts in the particular fields, and the cost of industrial production. Although nanotechnology has had a major contribution in medicine, particularly in cancer therapy, a common nanotoxicology consensus remains undefined [50]. For instance, long-term toxicity and safety profiles associated with nanoparticles need to be determined, but so far acute toxicity have largely been explored on a case-by-case basis. Specifically, the molecular interactions between nanocarriers and the endothelial cell lining should be unraveled, as most of these drug formulations are administered intravenously. In order to minimize the systemic toxicity, certain design principles can be considered, such as overall size and physical and chemical surface properties, as well as the selection and tailoring of the nanoformulations [101]. In the best-case scenario, functionalizing the nanocarrier with targeting ligands should help to reduce side effects by selectively increasing the drug accumulation at target sites and thereby reducing systemic exposure, eventually improving the therapeutic outcome [102]. Additionally, novel screening tests need to be designed to assess the biodistribution and understand the biochemical pathways that regulate cell functions. Although liposome-based nanoformulations are frequently used due to their non-immunogenicity, their short half-life, low solubility, and high production costs are to their disadvantage [103]. Polymeric-based nanoformulations have the advantage of being biodegradable, but at the same time, their drug release takes place in an uncontrolled manner, which is disadvantageous from an optimal nanoformulation point of view [104,105]. Moreover, there is a need to consider the cost of production for these nanoformulations for clinical use, as the cost of these combined chemo-antiangiogenic nanoformulations may supposedly be higher than the total cost of each drug altogether. The potential solution to the above-mentioned challenges lies in bringing the interdisciplinary experts and collaborators from academics, clinicians, scientists, and regulatory bodies to ensure a goal to establish the improved therapeutic outcomes of combined chemo-antiangiogenesis therapy and, ultimately, improving the quality of life of cancer patients.

Furthermore, the exploration of nanoformulations comprising anticancer drugs in combination with anti-inflammatory drugs [106,107,108], radioligands [109,110,111,112] and specific target genes is highly warranted [113]. These approaches would be beneficial in improving the therapeutic outcome, in addition to studies addressing the long-term toxicity and safety of the nanoformulations. Different biodegradable linkers can also be engineered to deliver the parent drugs through a controllable and targetable fashion, with the drug released sequentially or simultaneously via specific mechanisms in the tumor microenvironment, with the potential to achieve great advances in cancer therapy. Moreover, the incorporation of imaging agents (e.g., radionuclides or inorganic constructs) would also allow clinicians to tailor combination therapies in a more personalized manner and/or for complex cancers such ovarian cancer [114,115]. In conclusion, nanoformulations based on different nanocarriers, such as polymeric nanoparticles, micelles, hydrogels, liposomes, mesoporous silica nanoparticles, and gold nanoparticles containing anticancer and antiangiogenic agents have been established as a promising cancer treatment strategy in preclinical models. Nevertheless, there is still a long road for clinical application in terms of patient outcomes; however, these approaches offer flexible and robust platforms for the realization of the presented combination therapy concepts down the road.

## Figures and Tables

**Table 1:** Recently developed nanoformulations of anticancer and antiangiogenic drugs for combination cancer therapy.

Type of Nanoformulations	Nanocarrier Materials	Combination Drugs	Encapsulation Method	Cancer Types (*In Vivo*)	Outcome	Ref
**Polymer based**						
Polymeric nanoparticles	*D-*a-tocopheryl poly-ethylene glycol 1000-block-poly(*b-*amino ester) polymers	Doxorubicin and Curcumin	Physical encapsulation + Physical encapsulation	Hepatocellular carcinoma (human tumour xenograft model)	pH-sensitive nano-carrier enhanced the synergistic effect of simultaneous delivery of Dox and Cur	[80]
Polymer-self-assembled nanoparticles	Polyethylene glycol-vitamin E succinate	Curcumin and Sorafenib	Intermolecular hydrophobic interactions	Hepatocellular carcinoma (human tumour xenograft model)	Co-assembled nanoparticles provided higher therapeutic efficacy against tumor progression compared with free drug monotherapy or their free combination	[81]
Polymeric nanoparticles	Methoxypoly (ethylene glycol)-block-poly(*d,l-*lactide) copolymer and poly(acrylic acid*-co-*4-vubylphenylbornonic acid	7-ethyl-10-hydroxycamptothecin (SN38) and Combretastatin-A4	Chemical conjugation + Chemical conjugation	Colon cancer (human tumour tumors model)	Synergistic antiproliferative and antiangiogenic effects observed	[82]
Hydrogel	Poly(*N-(*3,4- dihydroxyphenethyl) methacrylamide-*co*-polyethylene glycol methyl ether methacrylate	Doxorubicin and Combretastatin-A4 Phosphate	Electrostatic + Physical encapsulation	Hepatocellular carcinoma (HepG2 xenograft model)	Sequential local delivery with superior *in vivo* efficacy observed	[83]
Micelle	Polyethylene glycol	Camptothecin and Combretastatin A4	Chemical conjugation + Chemical conjugation	-	Enhanced anticancer activity with a strong synergistic effect	[84]
Polymeric-self- assemble nanoparticles	Cyclo Arg-Gly-Asp-d-Tyr-Lys and low molecular weight heparin	Gambogic acid and cyclo (Arg-Gly-Asp-D-Tyr-Lys) peptide	Chemical conjugation	Glioblastoma (tumour xenograft model)	Efficiently inhibited the tumor growth in xenograft model with a reduced side-toxicity	[85]
Polymer-drug conjugate	*N*-(2-hydroxypropyl)meth acrylamide copolymer	Paclitaxel and Alendronate	Chemical conjugation + Chemical conjugation	Breast Cancer Bone Metastasis (4T-mCherry adenocarcinoma model)	Improved antitumor and antiangiogenic activity observed	[86]
Micelle	*α-*Aminopropyl-ω-methoxy-poly(ethylene glycol) and *β-*benzyl*-l-*aspartate *N-*carboxyanhydride	Paclitaxel and Rapamycin	Chemical conjugation + Chemical conjugation	Ovarian Cancer (ES2 murine xenograft model)	Synergistic apoptotic and antiangiogenic effects observed	[87]
Micelle	Poly(ethylene glycol)*-b-*poly(*d,l-*lactide)	Doxorubicin and Combretastatin-A4	Physical encapsulation + Physical encapsulation	Skin and lung cancer (B16-F10 tumor-bearing mice)	Exhibited stronger tumor growth inhibition and greater survival rate compared with the other treatment groups	[88]
**Lipid based**						
Nanocell	Poly(lactic-*co*-glycolic acid)copolymer	Doxorubicin and Combretastatin-A4	Chemical conjugation + Physical encapsulation	Skin and lung cancer (B16/F10 melanoma and Lewis lung carcinoma	Improved therapeutic index with reduced toxicity	[89]
Liposome	Egg phosphatidylcholine + 1,2-distearoyl-sn-glycero-3-phosphoethanolamine*-N*-[ amino(polyethylene glycol) +Cholesterol	Doxorubicin and Combretastatin-A4	Physical encapsulation + Physical encapsulation	Breast cancer (tumor bearing xenografts model)	Programmed *in vitro* drug release observed with sequential *in vivo* Cytotoxic effects of these two drugs	[90]
Liposome	Egg phosphatidylcholine + 1,2-distearoyl-sn-glycero-3-phosphoethanolamine *N-*[ amino(polyethylene glycol)] + Cholesterol + arginine-glycine-aspartic acid	Doxorubicin and Combretastatin-A4	Physical encapsulation + Physical encapsulation	B16F10 melanoma (C57BL/6 xenografts model)	Synergistic effect of the combined therapeutics with the increased anti-tumor response observed	[91]
**Inorganic based**						
Super magnetic iron oxide nanoparticles	Super magnetic iron oxide and albumin/bovine serum albumin	Curcumin and Sunitinib	Physical encapsulation + Physical encapsulation	Breast cancer (MCF-7 xenograft mouse model)	Observed significant tumor inhibition with a reduced systemic toxicity	[92]
Mesoporous silica nanoparticle	Cetyltrimethylammonium chloride tetraethyl orthosilicate +arginine-glycine-aspartic acid	Doxorubicin and Combretastatin-A4	Physical encapsulation + Physical conjugation	Human cervical adenocarcinoma (HeLa xenograft model)	Observed synergistic anticancer and antiangiogenic effected	[93]
Gold nanoparticles	Thiol polyethylene glycol/folic acid-tethered thiol polyethylene glycol and polydopamine-coated gold	Doxorubicin and dopamine	Electrostatic interactions	Breast cancer (MCF-7/Adriamycin resistance cells) tumor xenograft model	Superior tumor inhibitory effects against multidrug resistance cancer	[94]
